# Osthole Protects against Acute Lung Injury by Suppressing NF-*κ*B-Dependent Inflammation

**DOI:** 10.1155/2018/4934592

**Published:** 2018-07-03

**Authors:** Yiyi Jin, Jianchang Qian, Xin Ju, Xiaodong Bao, Li Li, Suqing Zheng, Xiong Chen, Zhongxiang Xiao, Xuemei Chen, Weiwei Zhu, Weixin Li, Wencan Wu, Guang Liang

**Affiliations:** ^1^Chemical Biology Research Center, School of Pharmaceutical Sciences, Wenzhou Medical University, Wenzhou, Zhejiang, China; ^2^The Eye Hospital of Wenzhou Medical University, Wenzhou, Zhejiang, China; ^3^Department of Cardiology, the First Affiliated Hospital, Wenzhou Medical University, Wenzhou, Zhejiang, China; ^4^Department of Pharmacy, Affiliated Yueqing Hospital, Wenzhou Medical University, Wenzhou, Zhejiang, China

## Abstract

Inflammation is a key factor in the pathogenesis of ALI. Therefore, suppression of inflammatory response could be a potential strategy to treat LPS-induced lung injury. Osthole, a natural coumarin extract, has been reported to protect against acute kidney injury through an anti-inflammatory mechanism, but its effect on ALI is poorly understood. In this study, we investigated whether osthole ameliorates inflammatory sepsis-related ALI. Results from *in vitro* studies indicated that osthole treatment inhibited the LPS-induced inflammatory response in mouse peritoneal macrophages through blocking the nuclear translocation of NF-*κ*B. Consistently, the *in vivo* studies indicated that osthole significantly prolonged the survival of septic mice which was accompanied by inflammation suppression. In the ALI mouse model, osthole effectively inhibited the development of lung tissue injury, leukocytic recruitment, and cytokine productions, which was associated with inhibition of NF-*κ*B nuclear translocation. These findings provide evidence that osthole was a potent inhibitor of NF-*κ*B and inflammatory injury and suggest that it could be a promising anti-inflammatory agent for therapy of septic shock and acute lung injury.

## 1. Introduction

Sepsis is a systemic and deleterious inflammatory response elicited by microbial infection [1–3]. The number of reported cases of sepsis continues to increase by 5 ~ 10% each year [4], making it one of the leading causes of death in intensive care facilities [5]. Among these case reports, approximately 30% of patients progress to multiorgan dysfunction syndrome (MODS) with 18% developing acute lung injury (ALI) [6–8]. ALI is a severe form of diffuse lung disease described as a clinical syndrome of acute respiratory failure with high morbidity and mortality [9]. It is characterized with persistent pulmonary inflammation [10] and increase in microvascular permeability [11]. Even with patients surviving ALI, the quality of life remains poor. Therefore, there is a great need for more effective therapeutic approaches.

Endotoxin, especially lipopolysaccharide (LPS) [12], is well recognized in the pathogenesis of ALI. LPS is a potent activator of toll-like receptor 4 (TLR4) [13–16], triggering the nuclear factor-kappa B (NF-*κ*B) pathway, thereby producing proinflammatory molecules, such as cytokines interleukin 6 (IL-6) [17], IL-1b [18], and tumor necrosis factor *α* (TNF-*α*) [19]. Excessive inflammatory responses induced by endotoxin can lead to tissue destruction, fibrosis, and eventual organ failure [4, 18, 20]. Therefore, blocking inflammatory cascades is considered an effective strategy to attenuate lung injury. However, current effective therapeutic agents remain inadequate.

Osthole, a natural coumarin extract from the fruit of *Cnidium monnieri* (L.) [21], has several beneficial pharmacological properties, such as antiseizure [22], antiosteoporosis [23], and antitumour [24] activities. Recently, studies found that osthole attenuates chronic kidney failure by inhibition of inflammation [25]. It also suppresses acute inflammatory responses in acute mechanical brain injury and kidney injury [26–28]. However, its potential protective effects on sepsis and ALI are not well characterized. In the present study, we evaluated the protective effects of osthole on LPS-induced ALI and its underlying mechanism. Our findings would provide important insight on potential new therapeutic approaches for inflammation-related lung injury.

## 2. Materials and Methods

### 2.1. Reagents

**Figure 1:**
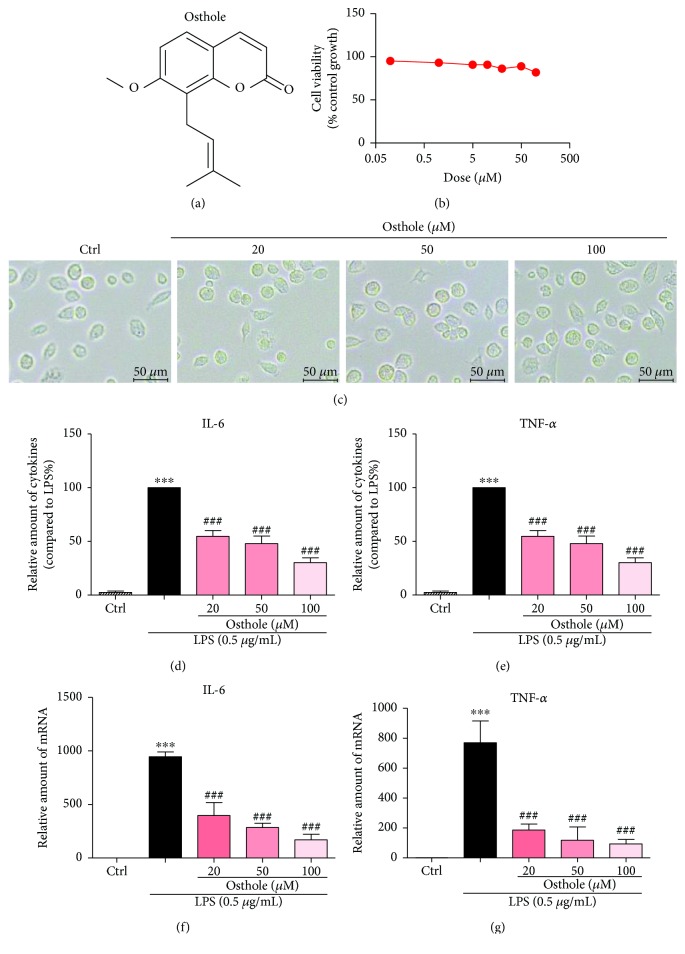
Osthole inhibits LPS-induced inflammatory cytokines in mouse peritoneal macrophages (MPMs). (a) The chemical structure of osthole. (b) MPMs were treated with various concentrations of osthole for 24 h. The viability of cells was measured by MTT assay and values reported as mean ± SEM; *n* = 3. (c) MPMs were treated with osthole (20, 50, or 100 *μ*M) for 24 h, and light micrographs were recorded using a microscope. Representative of three evaluations, *n* = 3, micron bar = 20 *μ*m. (d–e) MPMs were pretreated with osthole (20, 50, or 100 *μ*M) for 0.5 h and stimulated with LPS (0.5 *μ*g/mL) for 24 h, and secreted IL-6 (d) and TNF-*α* (e) were measured from conditioned media by ELISA (Materials and Methods). Values normalized to total protein of cells and reported as mean ± SEM relative to LPS alone; *n* = 3. (f–g) MPMs were pretreated with osthole (20, 50, or 100 *μ*M) for 0.5 h and stimulated with LPS (0.5 *μ*g/mL) for 6 h, and total RNA was extracted for real-time qPCR for IL-6 (f) and TNF-*α* (g). Values normalized to *β*-actin mRNA and reported as mean ± SEM; *n* = 3. (d–g) ^∗∗∗^*p* < 0.001 versus control; ^###^*p* < 0.001 versus LPS alone.

Osthole was purchased from Aladdin (Shanghai, China). The chemical structure is shown in Figure 1(a). Osthole was dissolved in dimethyl sulfoxide (DMSO) as 100 mM stock and diluted before use in assays. The final concentration of DMSO did not exceed 0.1%. All other reagents not mentioned were obtained from Sigma unless otherwise specified.

### 2.2. Cell Culture and Treatment

Mouse peritoneal macrophages (MPMs) were prepared as follows and also described in our previous paper [29]. Briefly, each ICR mice (8 weeks) was stimulated by intraperitoneal (ip) injection of 3 mL 6% thioglycollate solution (beef extract (0.3 g), tryptone (1 g), sodium chloride (0.5 g), and soluble starch (6 g)) dissolved in 100 mL water and filtrated with 0.22 *μ*m filter. Three days later, MPMs were harvested by washing the peritoneal cavity with 8 mL PBS containing 30 mM of EDTA. The suspension was centrifuged at 4°C, 1000 rpm, resuspended in RPMI-1640 (Gibco/BRL life Technologies, Eggenstein, Germany) with 10% (*v*/*v*) FBS (HyClone, Logan, UT, USA), 100 U/mL penicillin G, and 100 mg/mL streptomycin. Cells were cultured in a 37°C, 5% CO_2_ incubator, washed with medium 3 h after incubation, and used for studies after adherence firmly to culture plates.

GAPDH, P65, I*κ*B-*α*, and lamin B antibodies were purchased from Santa Cruz Biotechnology (Santa Cruz, CA, USA). TNF-*α* and CD68 antibodies were obtained from Abcam (Abcam, USA). The secondary antibody was purchased from Santa Cruz Biotechnology.

### 2.3. Immunofluorescence and Immunoblotting

MPMs were plated (1 × 10^6^) into a 6-well plate, pretreated with osthole for 1 h, and stimulated with LPS at indicated concentrations and time. After treatment, the cells were fixed with 4% paraformaldehyde and permeabilized with 100% methanol at 4°C for 5 min. The cells were washed twice with PBS containing 1% BSA and incubated with primary antibodies for anti-P65 antibody (1 : 200) overnight at 4°C, followed by a PE-conjugated secondary antibody (1 : 200). The cells were counterstained with DAPI and viewed by a Nikon fluorescence microscope (200x amplification, Nikon, Japan).

For Western blot analysis, the cells or lung tissue (30–50 mg) was prepared into homogenate samples and lysed (Boster Biological Technology, USA) and the protein concentration determined by a Bio-Rad protein assay kit (Bio-Rad, USA). Nuclear protein fractions were prepared using a kit from Beyotime (Shanghai, China) and were loaded and separated in 10% or 12% SDS-PAGE gels. The separated bands were electrotransferred to a nitrocellulose membrane and blocked in Tris-buffered saline, pH 7.6, containing 0.05% Tween 20 and 5% nonfat milk. Specific antibodies were incubated to probe for markers, and the immunoreactive bands were detected by incubating with a secondary antibody conjugated with horseradish peroxidase and visualized using enhanced chemiluminescence reagents (Bio-Rad, Hercules, CA).

### 2.4. Determination of TNF-*α* and IL-6 by ELISA

The TNF-*α* and IL-6 contents in culture medium or animal samples were determined by ELISA according to the manufacturer's instructions (Bioscience, San Diego, CA). The amount of TNF-*α* and IL-6 was normalized to protein concentration of cells, weight of animal tissues, or serum volume.

### 2.5. Real-Time Quantitative PCR

Total RNA was isolated from 1 × 10^6^ cells or tissues (50–100 mg) using TRIzol (Life Technologies, Carlsbad, CA). Reverse transcription and quantitative PCR (RT-qPCR) were performed using M-MLV Platinum RT-qPCR Kit (Life Technologies). Real-time qPCR was carried out using the Eppendorf RealPlex 4 instrument (Eppendorf, Hamburg, Germany). Primers for genes (i.e., TNF-*α*, IL-6, and *β*-actin) were obtained from Life Technologies. The primer sequences used are shown in Table 1. The relative amount of each gene was normalized to *β*-actin.

### 2.6. Mouse Models

Male C57BL/6 mice weighing 18–22 g were obtained from the Animal Centre of Wenzhou Medical University (Wenzhou, China). The mice were housed at constant room temperature with a 12 : 12 h light-dark cycle, fed a standard rodent diet and water, and acclimatized to the laboratory for at least 3 days before use for studies. All animal care and experimental procedures were approved by the Wenzhou Medical College Animal Policy and Welfare Committee.

#### 2.6.1. LPS-Induced Sepsis Model

Osthole was dissolved in 30% PEG 400 (Ludwigshafen, Germany), 1% volume of DMSO (Solarbio, Beijing, China), and 69% saline. Mice were pretreated with osthole (20 or 40 mg/kg) by ip injection, and 0.5 h later, 20 mg/kg LPS was injected through the tail vein. The mice were euthanized with chloral hydrate 6 h after LPS injection, and lung and liver tissues were excised aseptically, blotted dry, weighed, and immediately frozen in liquid nitrogen. The samples were stored at −80°C for later analyses. The body weight and mortality were recorded for 7 days.

#### 2.6.2. LPS-Induced ALI

The mice were randomly divided into four groups as follows: control (7 mice received vehicle of 0.9% saline), LPS (7 mice received LPS alone), 20 + LPS (7 mice received 20 mg/kg/day osthole and LPS), and 40 + LPS (7 mice received 40 mg/kg/day osthole and LPS). Osthole was a pretreatment given by ip injection for one week. LPS challenge was made by intratracheal injection of 50 *μ*L of LPS (5 mg/kg, dissolved in 0.9% saline) or 50 *μ*L 0.9% saline as vehicle control. Mice were euthanized with chloral hydrate 6 h after LPS injection, and bronchoalveolar lavage fluid (BALF) and blood samples were collected. Lung and liver tissues were excised aseptically, frozen in liquid nitrogen, and stored at −80°C before later analyses.

### 2.7. BALF Analysis

The collected BALF was centrifuged at 1000 rpm for 10 min at 4°C. The supernatant was used for protein concentration and cytokine determination. The cell pellet was resuspended using 50 *μ*L physiological saline for total cell count determination using a cell counting instrument (Count Star, Shanghai, China). The number of neutrophils in BALF was determined using Wright Giemsa staining (Nanjing Jiancheng Bioengineering Institute, Nanjing, China), and microscopic fields were counted under a Nikon fluorescence microscope (200x amplification; Nikon, Japan).

### 2.8. Lung Wet/Dry Ratio

For determining the ratio of wet to dry weight of lung tissue, the middle lobe of the right lung was collected and the wet weight was recorded. The tissue was heated at 65°C in a thermostatically controlled oven for 72 h and weighed. The ratio of wet/dry weight of lung tissue was reported as index of pulmonary edema.

### 2.9. Immunohistochemical Determination

Lung tissues were routinely fixed in 4% formalin, processed in graded alcohol and xylene, and embedded in paraffin. Paraffin blocks were sectioned into 5 *μ*m thick sections. After rehydration, the sections were stained with hematoxylin and eosin (H&E assay kit, Beyotime, Shanghai, China).

For immunohistochemistry, paraffin was removed from the sections with xylene, rehydrated in graded alcohol series, subjected to antigen retrieval in 0.01 mol/L citrate buffer (pH 6.0) by microwaving, and then placed in 3% hydrogen peroxide in methanol for 30 min at room temperature. After blocking with 5% BSA (Sigma, USA), the sections were incubated with anti-TNF-*α* antibody (1 : 500, Abcam, USA) or anti-CD68 (1 : 500, Abcam, USA) overnight at 4°C, followed by the secondary antibody (1 : 200; Santa Cruz, USA). The reaction was visualized with DAB (ZSGB-Bio, Beijing, China), counterstained with hematoxylin, dehydrated, and viewed under a Nikon fluorescence microscope (200x amplification, Nikon, Japan).

### 2.10. Statistical Analysis

All data represent three independent experiments and are expressed as means ± SEM. Statistical analyses were performed using GraphPad Pro Prism 5.0 (GraphPad, San Diego, CA). One-way ANOVA followed by multiple comparisons test with Bonferroni correction was used to analyze the differences between sets of data.

## 3. Results

### 3.1. Osthole Inhibits LPS-Induced Production of IL-6 and TNF-*α* in MPMs

The MTT assay was used to investigate the effect of osthole on cell viability. For study, MPMs were treated with several doses for 24 h. The results indicated that osthole did not impair cell viability at the range of concentrations used (Figure 1(b)) or alter cell morphology (Figure 1(c)). Therefore, we selected 20–100 *μ*M of osthole to investigate its anti-inflammatory activity. We first evaluated the effects of osthole on LPS-stimulated production of proinflammatory cytokines, IL-6 and TNF-*α*, in MPMs. Results indicated that LPS (0.5 *μ*g/mL) stimulation for 24 h of MPMs robustly increased the secretion of both IL-6 and TNF-*α* into medium (Figures 1(d) and 1(e), resp.). Pretreatment of MPMs with osthole for 0.5 h suppressed the LPS-induced cytokine secretion in a dose-dependent manner (Figures 1(d) and 1(e)). The osthole-induced inhibition of cytokine secretion was associated with inhibition of the transcription of IL-6 and TNF-*α* (Figures 1(f) and 1(g)). The results indicated that osthole can inhibit LPS-induced inflammatory response in MPMs.

### 3.2. Osthole Blocks LPS-Induced Activation of NF-*κ*B

**Figure 2:**
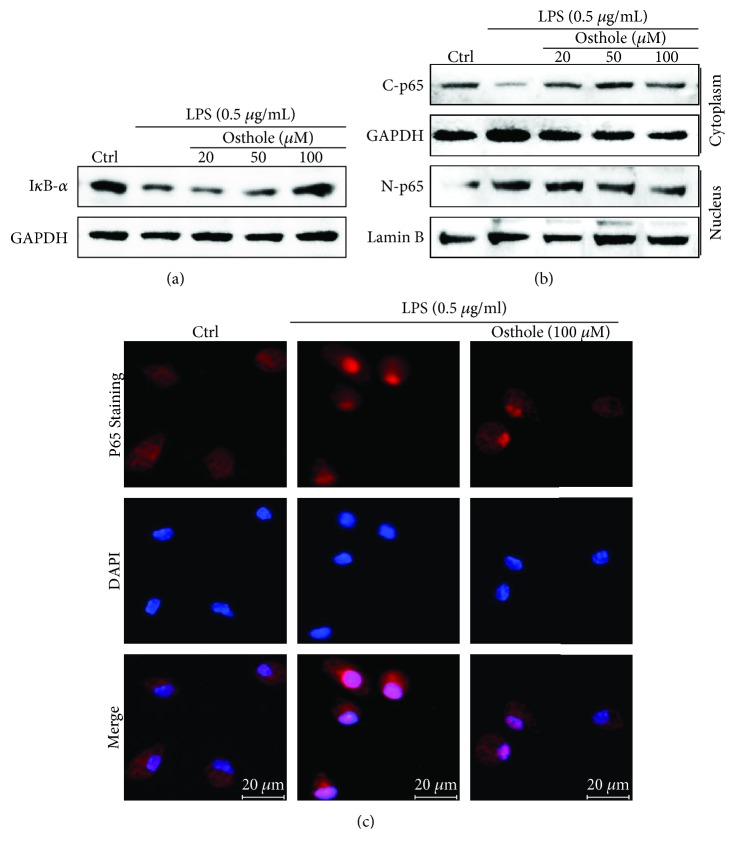
Osthole inhibits nuclear translocation of NF-*κ*B. The effects of osthole on LPS-activated NF-*κ*B were determined from MPMs pretreated with the indicated concentrations of osthole for 1 h and stimulated with LPS (0.5 *μ*g/mL) for 0.5 h (a–b) or 1 h (c). (a) Representative Western blot analysis of cell lysates for I*κ*B-*α*; GAPDH as loading control; *n* = 3. (b) Representative Western blot analysis of cytoplasmic and nuclear cell fractions for the p65 subunit of NF-*κ*B (C-P65 and N-P65, resp.); GAPDH and lamin B are respective loading controls; *n* = 3. (c) For immunofluorescence evaluation of nuclear translocation of the p65 subunit, the osthole-pretreated MPMs were stimulated with LPS (0.5 *μ*g/mL) for 1 h; p65 (red) and DAPI nuclear stain (blue), *n* = 3, micron bar = 20 *μ*m.

The production of proinflammatory cytokines is predominantly regulated by NF-*κ*B and/or AP-1 at the transcriptional level. MPMs were pretreated with osthole for 1 h and stimulated with LPS (0.5 *μ*g/mL) for 1 h, and cell lysates were immunoblotted for I*κ*B-*α* or the p65 subunit of NF-*κ*B. Results indicated that LPS significantly stimulated the degradation of I*κ*B-*α*, which was effectively prevented by osthole pretreatment in a dose-dependent manner (Figure 2(a)). The inhibition of the LPS-stimulated I*κ*B-*α* degradation was accompanied by inhibition of nuclear translocation of the p65 subunit of NF-*κ*B as detected by Western blot analysis of nuclear and cytoplasmic cell fractions (Figure 2(b)). Additionally, immunofluorescence staining of the p65 subunit similarly showed that osthole pretreatment prevented the LPS-induced nuclear translocation (Figure 2(c)). These data indicated that osthole suppressed LPS-induced activation of NF-*κ*B signaling.

### 3.3. Osthole Ameliorates LPS-Induced Sepsis *In Vivo*

**Figure 3:**
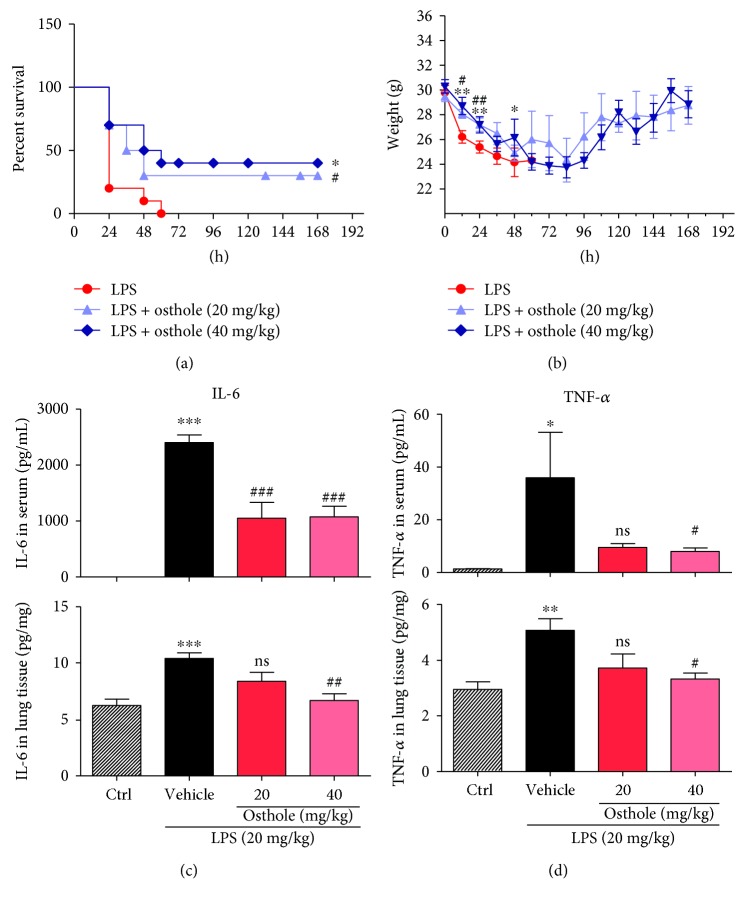
Osthole prolongs survival and inhibits inflammatory cytokines in LPS-induced septic mice. C57BL/6 mice (*n* = 10 each group) were pretreated for 0.5 h with 20 mg/kg or 40 mg/kg osthole via ip injection, followed by 20 mg/kg LPS by iv injection. (a) Percent survival for up to 168 h post-LPS injection is reported as mean ± SEM; *n* = 3; ^∗^, *p* < 0.05, versus control (Ctrl), and ^#^*p* < 0.05, versus LPS. (b) Body weight change was monitored for up to 168 h post-LPS injection and reported as mean ± SEM; *n* = 3; ^∗^*p* < 0.05 and ^∗∗^*p* < 0.01, versus Ctrl; ^#^*p* < 0.05 and ^##^*p* < 0.01, versus LPS. ELISA determination of (c) IL-6 and (b) TNF-*α* content in serum and lung tissue from septic mice; values are normalized to serum volume (c) or tissue weights (d) and reported as mean ± SEM; ns = not significant, ^∗^*p* < 0.05, ^∗∗^*p* < 0.01, and ^∗∗∗^*p* < 0.001, versus Ctrl. ^#^*p* < 0.05, ^##^*p* < 0.01, and ^###^*p* < 0.001, versus vehicle.

We used the LPS-induced sepsis mouse model to evaluate the protective effects of osthole. Mice given an iv injection of LPS died within 60 h (Figure 3(a)), as well as sharp loss of body weight (about 20%) (Figure 3(b)). However, mice pretreated with osthole, 20 or 40 mg/kg, improved survival beyond 72 h (*p* < 0.05) (Figure 3(a)), as well as body weight gain (Figure 3(b)). Uncontrollably sustained and vigorous inflammatory responses are characteristic of sepsis. In septic mice, both IL-6 and TNF-*α* were significantly elevated in serum and lung tissue (Figures 3(c) and 3(d)). Following osthole pretreatment, the increases in IL-6 and TNF-*α* content in serum and lung tissue were prevented (Figures 3(c) and 3(d)). The findings indicated that osthole was a potent inhibitor of inflammatory cytokine production, which likely protected against sepsis.

### 3.4. Protective Effect of Osthole on LPS-Induced Acute Lung Injury (ALI)

**Figure 4:**
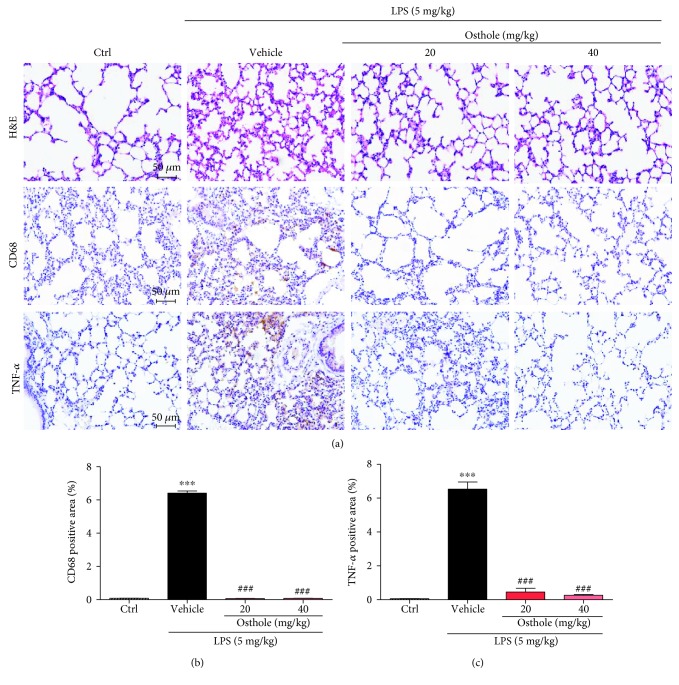
Osthole protects against inflammatory tissue injury in LPS-induced ALI. C57BL/6 mice were pretreated for 0.5 h with 20 mg/kg or 40 mg/kg osthole via ip injection, followed by intratracheal injection of 5 mg/kg LPS, and lungs were sampled 6 h later for analyses. (a, top panel) Representative light micrograph of lung histological changes stained with H&E, (a, middle panel) of immunolocalization of CD68, marker for macrophages (brown), and (a, bottom panel) of tissue localization of TNF-*α* (brown), micron bar = 50 *μ*m. Quantification of positive lung tissue staining for (b) CD68 and (c) TNF-*α* in (a); values are reported as % mean ± SEM; *n* = 4. ^∗∗∗^*p* < 0.001 versus control (Ctrl). ^###^*p* < 0.001 versus vehicle.

We next investigated the protective effects of osthole in the LPS ALI mouse model. Histological examination of the lung morphology stained with H&E indicated that LPS induced the expected pathologic changes, including areas of inflammatory infiltration, hemorrhage, interstitial edema, thickening of the alveolar wall, and lung tissue destruction (Figure 4(a), top panel). Moreover, LPS induced significant macrophage infiltration, as indicated by increased tissue localization of marker CD68 (Figures 4(a), middle panel and 4(b)), as well as increased tissue content of TNF-*α* (Figures 4(a), lower panel and 4(c)). However, osthole pretreatment effectively prevented the LPS-induced morphological derangements, as well as the induced increases in CD68 and TNF-*α* in lung tissue (Figures 4(a)–4(c)).

**Figure 5:**
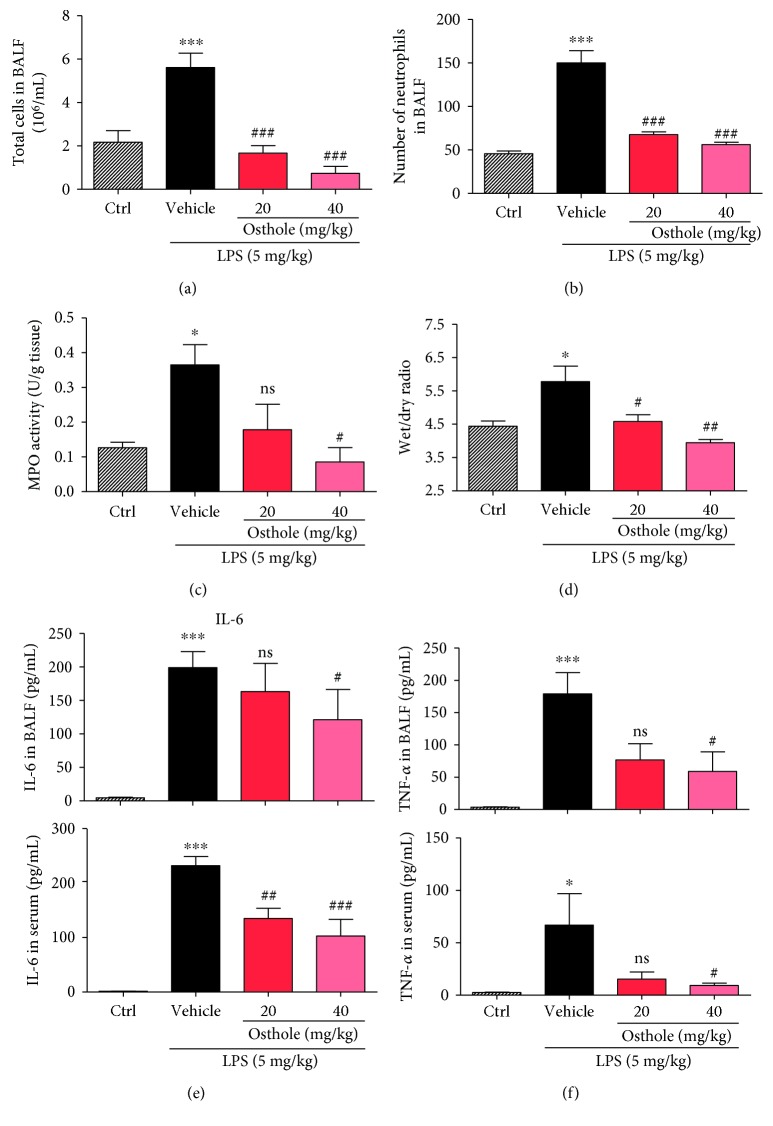
Osthole inhibits inflammatory cell infiltration and cytokine secretion in ALI. ALI was induced as described (Materials and Methods). Briefly, C57BL/6 mice were pretreated for 30 min with 20 mg/kg or 40 mg/kg osthole via ip injection, followed by intratracheal injection of 5 mg/kg LPS, and lungs were sampled 6 h later for analyses. (a) Number of total cells and (b) neutrophils in BALF was determined by counting microscopic fields; *n* = 7. (c) Lung tissue myeloperoxidase (MPO) activity assay was determined as an index of neutrophil activity; values are reported as mean ± SEM in U/g tissue; *n* = 7. (d) Wet and dry weights of lung tissue were measured and reported as the ratio of wet to dry as index of pulmonary edema; values are reported as mean ± SEM; *n* = 7. ELISA detection of (e) IL-6 and (f) TNF-*α* in BALF (upper graph) and serum (lower graph) from the experimental mice was determined, and values are reported as mean ± SEM; *n* = 7. ns = not significant; ^∗^*p* < 0.05 and ^∗∗∗^*p* < 0.001, versus control (Ctrl). ^#^*p* < 0.05, ^##^*p* < 0.01, and ^###^*p* < 0.001, versus vehicle.

Additionally, osthole pretreatment inhibited the LPS-induced extravasation and tissue recruitment of leukocytes in ALI. Results indicated that osthole inhibited the LPS-induced increase in total cell number and neutrophils in BALF (Figures 5(a) and 5(b)). This was further corroborated by the finding that the LPS-induced increase in lung tissue MPO activity, an index of neutrophil, was significantly inhibited by osthole pretreatment (Figure 5(c)). An important characteristic of ALI is pulmonary edema, which can be assessed by the wet/dry lung weight. LPS induced ~30% increase in the wet/dry ratio, indicating the presence of pulmonary edema, which was prevented by osthole pretreatment (Figure 5(d)). Further, LPS induced increases of proinflammatory cytokines IL-6 and TNF-*α*, in both BALF and serum (Figures 5(e) and 5(f)). As expected, osthole pretreatment inhibited these increases in BALF and serum (Figures 5(e) and 5(f)). These results provide strong evidence that osthole was a potent anti-inflammatory agent with therapeutic potential for treatment of ALI.

### 3.5. Osthole Suppresses the NF-*κ*B Nuclear Translocation in ALI Mouse Model

**Figure 6:**
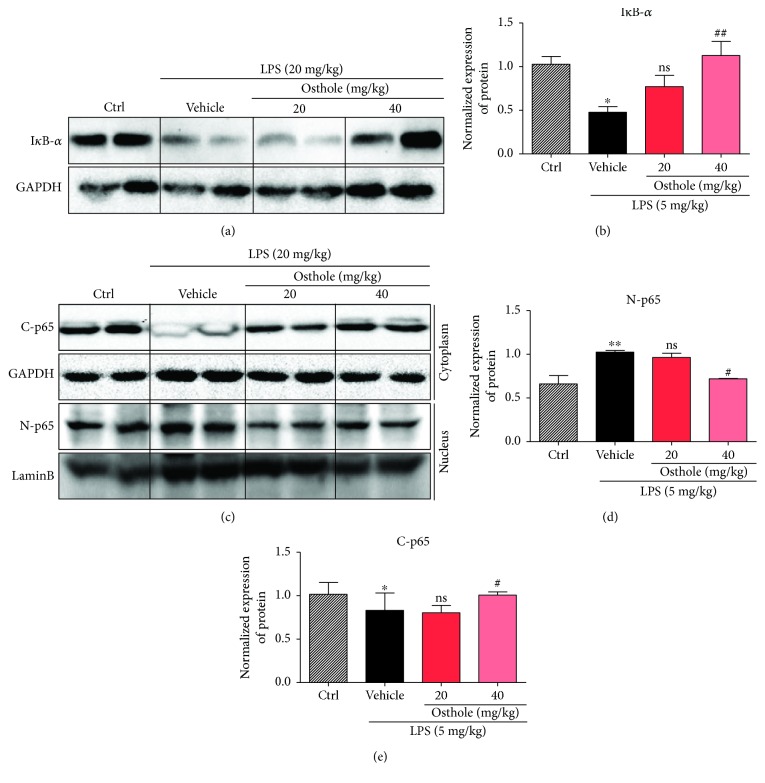
Osthole suppresses NF-*κ*B signaling *in vivo*. ALI was induced as described (Materials and Methods). Briefly, C57BL/6 mice were pretreated for 30 min with 20 mg/kg or 40 mg/kg osthole via ip injection, followed by intratracheal injection of 5 mg/kg LPS, and lungs were sampled 6 h later for Western blot analyses. (a) Representative Western blot detecting for I*κ*B-*α*; GAPDH as loading control. (b) Densitometric quantification of blot in (a); values are reported as mean ± SEM relative to control (Ctrl); *n* = 4. The bands were quantified by ImageJ and normalized to loading controls. (c) Representative Western blot analysis of the p65 subunit of NF-*κ*B from cytoplasmic and nuclear cell fractions (C-p65 and N-p65, resp.); GAPDH and lamin B as respective loading controls. Densitometric quantification of (d) N-p65 and (e) C-p65 from (c); values are reported as mean ± SEM; *n* = 4. The bands were quantified by ImageJ and normalized to loading controls. ns = not significant; ^∗^*p* < 0.05, ^∗∗∗^*p* < 0.001 versus control (Ctrl). ^#^*p* < 0.05, ^##^*p* < 0.01 versus vehicle.

We investigated the signaling mechanism by which osthole protected against inflammatory injury responses observed in our *in vivo* and *in vitro* studies. The involvement of NF-*κ*B was first evaluated by determining I*κ*B-*α* degradation in the mouse lung tissues of ALI mice. Western blot results indicated that LPS induced significant I*κ*B-*α* degradation, which was effectively prevented by osthole pretreatment (Figures 6(a) and 6(b)). Moreover, LPS stimulated the nuclear translocation of the p65 subunit of NF-*κ*B, but osthole pretreatment prevented the translocation (Figures 6(c)–6(d)). The data illustrated that osthole inhibited NF-*κ*B signaling in lung tissue of ALI *in vivo*.

## 4. Discussion

ALI is characterized by persistent pulmonary inflammation [10] and increase in microvascular permeability. Enhanced inflammatory responses [30, 31], manifesting as elevated inflammatory cytokine production, and macrophage infiltration in lung are the most important pathological mechanisms [32]. Therefore, anti-inflammatory therapy could be an attractive option to improve the quality of life in ALI patients. However, the progress in the development of novel effective therapeutic drugs for treatment of ALI is still disappointing. Discovery of active compounds from natural products would speed up the pace presently. Interestingly, several natural compounds with anti-inflammatory activities have been demonstrated to prevent inflammatory responses in experimental animal models of ALI.

Osthole has been shown to be effective in acute kidney injury via suppressing inflammatory response. However, the underlying mechanism is poorly understood. Here, we show that osthole alleviated ALI by inhibiting LPS-induced productions of IL-6 and TNF-*α*, likely through a mechanism in modulating NF-*κ*B activity. It was closely related to the efficacy of osthole against ALI, such as lung tissue derangements, pulmonary edema, tissue recruitment of leukocytes, and prolonging of the survival time of septic mice. Although the dose of osthole was relatively high, no toxic effects on body weight and cell proliferation were observed in our study. In addition, the effective dose of osthole we used was consistent with literatures reported previously [33, 34]. Furthermore, osthole is a natural coumarin derivative. Coumarin has been approved for some medical uses as pharmaceuticals in the treatment of lymphedema and anticoagulation via its anti-inflammatory properties. Thus, the present study would also provide a basis for expanding the indications of coumarin.

Macrophages are important participants in immune response [35–37] by providing an immediate defense against foreign agents or organisms. Besides, neutrophil infiltration into inflamed and infected tissues is a fundamental process of the innate immune response [38–41], which increased recruitment by the stimulation of LPS as the data shows. Two transcription factors, NF-*κ*B [42–44] and AP-1 [45], are well-characterized regulators in the expression of proinflammatory cytokines [46], including TNF-*α*, IL-6, IL-1*β*, and IL-18 [47]. Our findings indicated that the inhibition of inflammatory response by osthole was through suppressing I*κ*B-*α* degradation, resulting in NF-*κ*B inhibition and thereby inhibition of its downstream cytokine gene expression. However, osthole showed no significant effect on JNK and p38 MAPK signaling pathways (data not shown). Overall, our results indicated that osthole potently inhibited LPS-activated NF-*κ*B signaling of macrophages in the lung tissue of ALI mice, leading to significant suppression of inflammation and tissue injury. Even though nonsteroidal or steroidal anti-inflammatory drugs have potent anti-inflammation efficacy, the underlying mechanisms and side effects limit their applications in clinic. So, the present study would provide a potential strategy for treating ALI.

In summary, in this study we examined the potential pharmacological effect of osthole on sepsis and ALI and its underlying mechanism. Our work highlights osthole as a potential new candidate for treatment of injurious inflammatory responses in sepsis and ALI. However, the direct target of osthole upstream NF-*κ*B remains unknown. The family of toll-like receptors (TLRs) are know as initiator of innate immune response. TLR4 is a typical member of TLRs which responsible for chronic and acute inflammatory disorders. Moreover, TLR4 is the primary receptor for LPS. Future studies are needed to investigate the relationship between osthole and TLR4.

## Figures and Tables

**Table 1:** Primers used for real-time qPCR assay.

Gene	Species	FW (5′-3′)	RW (5′-3′)
TNF-*α*	Mouse	TGATCCGCGACGTGGAA	ACCGCCTGGAGTTCTGGAA
IL-6	Mouse	GAGGATACCACTCCCAACAGACC	AAGTGCATCATCGTTGTTCATACA
*β*-Actin	Mouse	CCGTGAAAAGATGACCCAGA	TACGACCAGAGGCATACAG

## References

[1] Wang J., Wu H., Yang Y. (2018). Bacterial species-identifiable magnetic nanosystems for early sepsis diagnosis and extracorporeal photodynamic blood disinfection. *Nanoscale*.

[2] Baggs J., Jernigan J. A., Halpin A. L., Epstein L., Hatfield K. M., McDonald L. C. (2018). Risk of subsequent sepsis within 90 days after a hospital stay by type of antibiotic exposure. *Clinical Infectious Diseases*.

[3] Abbasi M., Greenstein Y., Koenig S. (2017). Usefulness of ultrasound to help solve severe sepsis. *Chest*.

[4] Delano M. J., Ward P. A. (2016). The immune system’s role in sepsis progression, resolution, and long-term outcome. *Immunological Reviews*.

[5] Fattahi F., Ward P. A. (2017). Understanding immunosuppression after sepsis. *Immunity*.

[6] Krupa A., Fudala R., Florence J. M. (2013). Bruton’s tyrosine kinase mediates Fc*γ*RIIa/Toll-like receptor–4 receptor crosstalk in human neutrophils. *American Journal of Respiratory Cell and Molecular Biology*.

[7] Sun X., Singleton P. A., Letsiou E. (2012). Sphingosine-1–phosphate receptor–3 is a novel biomarker in acute lung injury. *American Journal of Respiratory Cell and Molecular Biology*.

[8] Liao Y., Song H., Xu D. (2015). *Gprc5a*-deficiency confers susceptibility to endotoxin-induced acute lung injury via NF-*κ*B pathway. *Cell Cycle*.

[9] Walkey A. J., Wiener R. S. (2012). Macrolide antibiotics and survival in patients with acute lung injury. *Chest*.

[10] Filgueiras L. R., Martins J. O., Serezani C. H., Capelozzi V. L., Montes M. B. A., Jancar S. (2012). Sepsis-induced acute lung injury (ALI) is milder in diabetic rats and correlates with impaired NFkB activation. *PLoS One*.

[11] Thamphiwatana S., Angsantikul P., Escajadillo T. (2017). Macrophage-like nanoparticles concurrently absorbing endotoxins and proinflammatory cytokines for sepsis management. *Proceedings of the National Academy of Sciences of the United States of America*.

[12] Rosadini C. V., Kagan J. C. (2017). Early innate immune responses to bacterial LPS. *Current Opinion in Immunology*.

[13] Beutler B. (2000). Tlr4: central component of the sole mammalian LPS sensor. *Current Opinion in Immunology*.

[14] Jang J. C., Li J., Gambini L. (2017). Human resistin protects against endotoxic shock by blocking LPS–TLR4 interaction. *Proceedings of the National Academy of Sciences of the United States of America*.

[15] Peri F., Piazza M. (2012). Therapeutic targeting of innate immunity with Toll-like receptor 4 (TLR4) antagonists. *Biotechnology Advances*.

[16] Yin J., Michalick L., Tang C. (2016). Role of transient receptor potential vanilloid 4 in neutrophil activation and acute lung injury. *American Journal of Respiratory Cell and Molecular Biology*.

[17] Zhang H., Neuhöfer P., Song L. (2013). IL-6 trans-signaling promotes pancreatitis-associated lung injury and lethality. *The Journal of Clinical Investigation*.

[18] Martínez-González I., Roca O., Masclans J. R. (2013). Human mesenchymal stem cells overexpressing the IL-33 antagonist soluble IL-1 receptor–like–1 attenuate endotoxin-induced acute lung injury. *American Journal of Respiratory Cell and Molecular Biology*.

[19] Patel B. V., Wilson M. R., O'Dea K. P., Takata M. (2013). TNF-induced death signaling triggers alveolar epithelial dysfunction in acute lung injury. *Journal of Immunology*.

[20] Ma S. F., Xie L., Pino-Yanes M. (2011). Type 2 deiodinase and host responses of sepsis and acute lung injury. *American Journal of Respiratory Cell and Molecular Biology*.

[21] Hoult J. R. S., Payá M. (1996). Pharmacological and biochemical actions of simple coumarins: natural products with therapeutic potential. *General Pharmacology*.

[22] Jarząb A., Grabarska A., Skalicka-Woźniak K., Stepulak A. (2017). Pharmacological features of osthole. *Postępy Higieny i Medycyny Doświadczalnej*.

[23] Wang P., Ying J., Luo C. (2017). Osthole promotes bone fracture healing through activation of BMP signaling in chondrocytes. *International Journal of Biological Sciences*.

[24] Wu C., Sun Z., Guo B. (2017). Osthole inhibits bone metastasis of breast cancer. *Oncotarget*.

[25] Wu H. X., Wang Y. M., Xu H. (2017). Osthole, a coumadin analog from *Cnidium monnieri* (L.) Cusson, ameliorates nucleus pulposus-induced radicular inflammatory pain by inhibiting the activation of extracellular signal-regulated kinase in rats. *Pharmacology*.

[26] Huang T., Dong Z. (2017). Osthole protects against inflammation in a rat model of chronic kidney failure via suppression of nuclear factor-*κ*B, transforming growth factor-*β*1 and activation of phosphoinositide 3-kinase/protein kinase B/nuclear factor (erythroid-derived 2)-like 2 signaling. *Molecular Medicine Reports*.

[27] Zhao X., Wang F., Zhou R., Zhu Z., Xie M. (2018). PPAR*α*/*γ* antagonists reverse the ameliorative effects of osthole on hepatic lipid metabolism and inflammatory response in steatohepatitic rats. *Inflammopharmacology*.

[28] Huang W. C., Liao P. C., Huang C. H., Hu S., Huang S. C., Wu S. J. (2017). Osthole attenuates lipid accumulation, regulates the expression of inflammatory mediators, and increases antioxidants in FL83B cells. *Biomedicine & Pharmacotherapy*.

[29] Wu J., Li J., Cai Y. (2011). Evaluation and discovery of novel synthetic chalcone derivatives as anti-inflammatory agents. *Journal of Medicinal Chemistry*.

[30] Lundbäck P., Lea J. D., Sowinska A. (2016). A novel high mobility group box 1 neutralizing chimeric antibody attenuates drug-induced liver injury and postinjury inflammation in mice. *Hepatology*.

[31] Hu Y., Lou J., Mao Y. Y. (2016). Activation of MTOR in pulmonary epithelium promotes LPS-induced acute lung injury. *Autophagy*.

[32] Vettorazzi S., Bode C., Dejager L. (2015). Glucocorticoids limit acute lung inflammation in concert with inflammatory stimuli by induction of SphK1. *Nature Communications*.

[33] Li Y., Li Y., Shi F., Wang L., Li L., Yang D. (2018). Osthole attenuates right ventricular remodeling via decreased myocardial apoptosis and inflammation in monocrotaline-induced rats. *European Journal of Pharmacology*.

[34] Yu C., Li P., Qi D. (2017). Osthole protects sepsis-induced acute kidney injury via down-regulating NF-*κ*B signal pathway. *Oncotarget*.

[35] Martinez F. O., Helming L., Gordon S. (2009). Alternative activation of macrophages: an immunologic functional perspective. *Annual Review of Immunology*.

[36] Chen K., Kolls J. K. (2013). T cell-mediated host immune defenses in the lung. *Annual Review of Immunology*.

[37] Kopf M., Schneider C., Nobs S. P. (2015). The development and function of lung-resident macrophages and dendritic cells. *Nature Immunology*.

[38] Kienle K., Lammermann T. (2016). Neutrophil swarming: an essential process of the neutrophil tissue response. *Immunological Reviews*.

[39] Favier B. (2016). Regulation of neutrophil functions through inhibitory receptors: an emerging paradigm in health and disease. *Immunological Reviews*.

[40] Hartl D., Lehmann N., Hoffmann F. (2008). Dysregulation of innate immune receptors on neutrophils in chronic granulomatous disease. *The Journal of Allergy and Clinical Immunology*.

[41] Martineau A. R., Newton S. M., Wilkinson K. A. (2007). Neutrophil-mediated innate immune resistance to mycobacteria. *The Journal of Clinical Investigation*.

[42] Covert M. W., Leung T. H., Gaston J. E., Baltimore D. (2005). Achieving stability of lipopolysaccharide-induced NF-*κ*B activation. *Science*.

[43] Baldwin A. S. (1996). The NF-*κ*B and I*κ*B proteins: new discoveries and insights. *Annual Review of Immunology*.

[44] Rao P., Hayden M. S., Long M. (2010). I*κ*B*β* acts to inhibit and activate gene expression during the inflammatory response. *Nature*.

[45] Wiesner P., Choi S. H., Almazan F. (2010). Low doses of lipopolysaccharide and minimally oxidized low-density lipoprotein cooperatively activate macrophages via nuclear factor *κ*B and activator protein-1: possible mechanism for acceleration of atherosclerosis by subclinical endotoxemia. *Circulation Research*.

[46] Jang S., Kelley K. W., Johnson R. W. (2008). Luteolin reduces IL-6 production in microglia by inhibiting JNK phosphorylation and activation of AP-1. *Proceedings of the National Academy of Sciences of the United States of America*.

[47] Li C. C., Munitic I., Mittelstadt P. R., Castro E., Ashwell J. D. (2015). Suppression of dendritic cell-derived IL-12 by endogenous glucocorticoids is protective in LPS-induced sepsis. *PLoS Biology*.

